# Effect of Fatigue Upon Performance and Electromyographic Activity in 6-RM Bench Press

**DOI:** 10.2478/hukin-2014-0007

**Published:** 2014-04-09

**Authors:** Roland van den Tillaar, Atle Saeterbakken

**Affiliations:** 1 Department of Teacher Education of Nord Trøndelag University College, Levanger Norway.; 2 Department of Teacher Education and Sports of Sogn and Fjordane University College, Sogndal, Norway.

**Keywords:** EMG, resistance exercise, trunk, coordination, kinematics

## Abstract

The aim of this study was to examine the effect of fatigue during one set of 6-RM bench pressing upon the muscle patterning and performance. Fourteen resistance-trained males (age 22.5±2.0 years, stature 1.82±0.07 m, body mass 82.0±7.8 kg) conducted a 6-RM bench press protocol. Barbell kinematics and EMG activity of pectoralis major, deltoid anterior, biceps brachii, triceps brachii, rectus abdominis, oblique external and erector spinae were measured in each repetition during the 6-RM bench press. Total lifting time increased and the velocity in the ascending movement decreased (p≤0.001). However, the kinematics in the descending phase deferred: the time decreased and velocity increased during the 6-RM (p≤0.001). Generally, muscles increased their EMG amplitude during the six repetitions in the ascending movement, while only three of the seven measured muscles showed an increase over the six repetitions in the descending part in 6-RM bench pressing. It was concluded that the bench pressing performance decreased (lower barbell velocities and longer lifting times) with increasing fatigue in the 6-RM execution. Furthermore EMG increased in the prime movers and the trunk stabilizers (abdominal and spine), while the antagonist muscle (biceps) activity was not affected by fatigue during the lifting phase in a single set of 6-RM bench pressing

## Introduction

In strength training for different sports and in weight lifting, the bench press is one of the most popular exercises for the upper body. In training often athletes carry out a number of sets at sub-maximal loads with several repetitions at a certain percentage of 1-RM to exhaustion. During these sets, fatigue is often experienced and sometimes the last repetition is completed with assistance.

This fatigue is recognized as a multifactorial phenomenon often shown in loss of force production and thereby visible as a loss of peak barbell velocity in the bench press (Drinkwater et al., 2007; Sanchez-Medina and Gonzalez-Badillo, 2011). Several studies are limited by investigating only the loss of power, force and velocity output during sets with sub-maximal loads (Duffey and Challis, 2009; Sanchez-Medina and Gonzalez-Badillo, 2011).

Sánchez-Medina and González-Badillo (2011) showed in resistance-trained subjects, decreased velocity in the 6-RM test until exhaustion and proposed that velocity loss can be an indicator for neuromuscular fatigue. However, no electromyographic muscle activity (EMG) was measured. Therefore, little is known about the muscle patterning during these repetitions. In the literature, conflicting results were found in which some studies showed increased muscle activation during fatigue in resistance exercises in experienced strength-trained subjects (Gentil et al., 2007; Brennecke et al., 2009), others showed a decreased EMG following maximal strength loading in healthy subjects (Häkkinen, 1993; Gerdle et al., 2000) or reported no EMG amplitude changes after fatigue (Lindström et al., 2006). Sub-maximal strength training under isokinetic conditions in youth males and females first demonstrated increased relative EMG amplitude followed by either a stable level or a decrease during 100 contractions (Lindström et al., 1997; Lindström et al., 2006). In these studies several contractions were conducted, which can be compared with strength endurance training (100 contractions) and not with regular maximal strength training. However, the application of isokinetic training studies to most athletic training appears questionable in terms of external validity (Abernethy et al., 1995). In strength training with free weights (isoinertial), Walker et al. (2012) showed that in the leg press, in subjects without resistance training experience during maximal strength training (15 sets of 1-RM) EMG decreased while with hypertrophic training (15 sets of 10-RM) EMG amplitude increased. Fatigue in the bench press had different effects on muscle activity as the pectoralis and deltoid muscles had similar EMG amplitude, while triceps muscle activity increased (Gentil et al., 2007; Brennecke et al., 2009). However, Brennecke et al. (2009) and Gentil et al. (2007) used experienced resistance-trained subjects and a pre-exhaustion exercise to investigate the effect of fatigue and not what occurred acute in muscle patterning during one set of the bench press. In addition 6-RM is often used in strength training to enhance maximal strength (Sanchez-Medina and Gonzalez-Badillo, 2011). Therefore the aim of this study was to examine the effect of fatigue during one set of 6-RM bench pressing upon the muscle patterning and performance (kinematics of the 6 repetitions) in experienced resistance-trained subjects. We expected increased EMG amplitude of the prime movers, while the stabilizing muscles did not increase their muscle activation due to the sub-maximal character of the bench press exercise. This sub-maximal character of the bench press in the start of the 6-RM gives the muscles the opportunity, when fatigued, to increase in firing amplitude.

## Material and Methods

### Participants

Fourteen resistance-trained males (age 22.5±2.0 years, stature 1.82±0.07 m, body mass 82.0±7.8 kg) with approximately 4.6±2.1 years of free weight strength training experience (including the bench press) volunteered for this study. The average load for the 6-RM bench press protocol equalled 85 ±15.6 kg. The 6-RM normalized to body weight was 1.05. Participants were excluded from the study if they had musculoskeletal pain, injury, illness that might reduce maximal effort or experienced pain during testing. All participants were familiar with the bench press exercise. The participants were instructed to refrain from any additional resistance training targeting the upper body during the 72 hours before testing. Ethics approval was obtained from the local research ethics committee and conformed to the latest revision of the Declaration of Helsinki and according to the latest ethical standards of this journal (Harriss and Atkinson, 2011). Each participant was informed of the testing procedures and possible risks, and written consent was obtained prior to the study.

### Procedures

In a familiarization session two weeks prior to the experimental test, the true six repetitions maximum (6-RM) load was tested and identified. During the test protocol, the head, shoulders and hips were supported by the bench with a ∼90° flexion in the knees. Each participant chose an optimal grip and feet position. Two spotters assisted the participants in the preload phase by lifting and stabilizing the Olympic barbell (2.8 cm diameter, length 1.92m) until the participant had fully extended arms. The barbell was lowered in a controlled manner, lightly touched the chest and lifted back to the starting position with fully extended elbows. No bouncing of the barbell was allowed. The participants were instructed to use a self-selected tempo in which they had full control over their lifting technique and performed with maximal effort.

Prior to the familiarization and 6-RM test, each participant performed a 10-min warm-up on a cycle ergometer or treadmill at an intensity corresponding to a rating of perceived exertion between 8 – 10 on the Borg scale (1998). Next, three warm-up sets were performed: 1) 20 repetitions at 25% of anticipated 1-RM, 10 repetitions at 50% of 1-RM and 8 repetitions at 70% of 1-RM (Behm et al., 2005). After the last warm-up set, the participants were asked to predict their 6-RM load. After six successful repetitions, the participants were asked if they thought the set was their 6-RM. If not, for each set heavier resistance was added so that their 6-RM could be identified. The 6-RM load was identified within one to three attempts. In general, a pause of 3–4 minutes was given between each trial in order to avoid fatigue. However, each subject decided himself when he felt ready (fully recovered from the last attempt) for a new attempt.

The EMG was measured from seven muscles: pectoralis major (approximately 4 cm medial to the axillary fold (Schick et al., 2010), anterior deltoid (1.5cm distal and anterior to the acromion), triceps brachii (long head, approximately 3 cm medial and on 50% on the line between acromion and olecranon), biceps brachii (1/3 from the fossa cubit), rectus abdominis (3 cm lateral to the umbilicus), oblique external (approximately 15 cm to the umbilicus) and erector spinae (L1, 6 cm lateral to the spinous process) according to the recommendations of SENIAM (Hermens et al., 2000) and as used in similar studies (Anderson and Behm, 2004; Behm et al., 2005). Before placement of the gel coated self-adhesive electrodes (Dri-Stick Silver circular sEMG Electrodes AE-131, NeuroDyne Medical, USA) the skin was shaved, washed with alcohol and abraded before the placement. The electrodes (11 mm contact diameter, 20 mm centre-to-centre distance) were placed on the side of the dominant arm (Behm et al., 2005; Saeterbakken and Fimland, 2011; van den Tillaar and Ettema, 2013).

EMG activity was measured with Musclelab 4020e (Ergotest Technology AS, Langesund, Norway). The raw EMG signals, sampled at 1000Hz were amplified and filtered using a preamplifier located as close to the pickup point as possible. The signals were high pass and low pass (600, 8 Hz) filtered, rectified, integrated and converted to root-mean-square (RMS) signals using a hardware circuit network (frequency response 450 kHz, averaging constant 12 ms, total error ± 0.5%). With a common rejection rate of 106 dB, the RMS signals were re-sampled at a rate of 100 Hz using a 16 bit A/D converter. A linear encoder (ET-Enc-02, Ergotest Technology AS, Langesund, Norway) connected to the barbell measured the lifting time of the descending and ascending part of the barbell of each repetition of the 6-RM test with a resolution of 0.075 mm and counts the pulses with 10 ms intervals (Arnason et al., 2004). Peak and average velocity of the barbell during the descending and ascending part was calculated using a five point differential filter with software Musclelab V8.13 (Ergotest Technology AS, Langesund, Norway). The linear encoder was synchronized with the EMG recordings using a Musclelab 4020e and analyzed by software V8.13 (Ergotest Technology AS, Langesund, Norway). The beginning and end of each of the six repetitions was identified and mean EMG RMS activities were calculated for the descending and ascending parts of each of the six repetitions (i.e. short stops at full arm extension were removed from the analysis).

### Statistical Analysis

To assess differences in kinematics and EMG activity during 6-RM testing, a One-way ANOVA with repeated measures (repetition: 1 to 6) was used with Holm-Bonferroni post-hoc tests. In cases where the sphericity assumption was violated, the Greenhouse-Geisser adjustments of the p-values were reported. The level of significance was set at *p*≤0.05. For statistical analysis purposes, the SPSS version 19.0 (SPSS, Inc., Chicago, IL) was applied. All results are presented as means ± standard deviations and effect size was evaluated with η^2^_p_ (Eta partial squared) where 0.01<η^2^<0.06 constitutes a small effect, a medium effect when 0.06<η^2^<0.14 and a large effect when η^2^>0.14 (Cohen, 1988).

## Results

**Figure 1.**
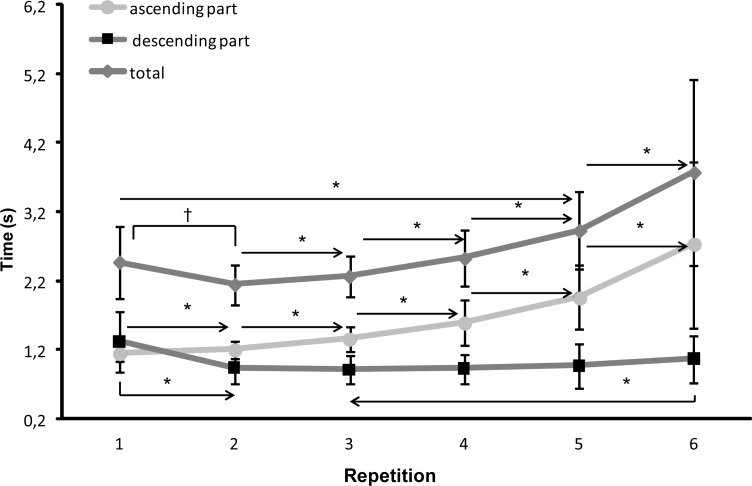
Mean (SD) in lifting time in the descending and ascending part together with the total lifting time of each repetition during 6-RM bench press. * indicates a significant difference between this repetition and all repetitions away from the sign, p ≤ 0.05 † indicates a significant difference between these two repetitions, p ≤ 0.05

**Figure 2.**
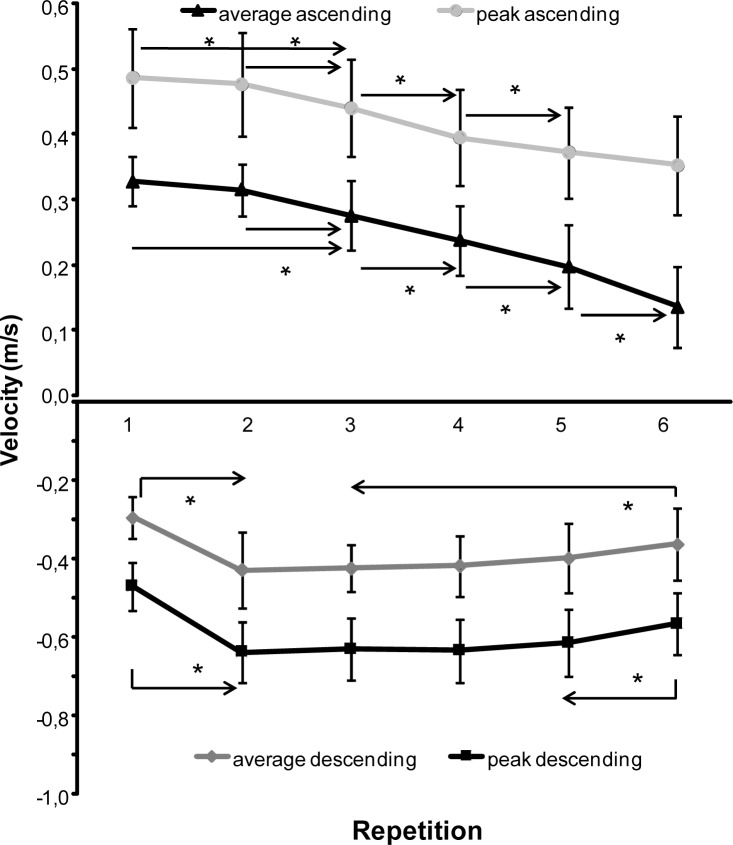
Mean (SD) peak and average velocity for each repetition in the descending and ascending part during 6-RM bench pressing. * indicates a significant difference between this repetition and all repetitions away from the sign, p ≤ 0.05 † indicates a significant difference between these two repetitions, p ≤ 0.05

The total lifting time for the 6-RM attempt was 15.89±2.25 s. A significant change in total lifting time (F=13.66, *p=*0.001 η^2^=0.53) was found from the first to the sixth repetition (Figure 1). Post hoc comparison showed that firstly the total lifting time decreased from repetition 1 to 2 and after that increased for every repetition. When the total lifting time was divided between the descending and ascending part, the lifting time in the descending part (F=9.47, *p*=0.001; η^2^=0.41) significantly decreased from repetition 1 to 2 and then only significantly increased again with repetition 6. In the ascending part (F=15.55, *p*=0.001; η^2^=0.55), the lifting time increased significantly in each repetition (Figure 1). In the descending part the velocity (F≥9.71, *p<*0.001; η^2^≥0.43) increased significantly from repetition 1 to 2 and was relatively stable from repetition 2, while it significantly increased again in repetition 6 (Figure 2). In the ascending part, the velocity of the barbell (F≥27.68, *p<*0.001; η^2^≥0.68) decreased significantly in each repetition from repetition 2 to 6 (Figure 2).

**Figure 3.**
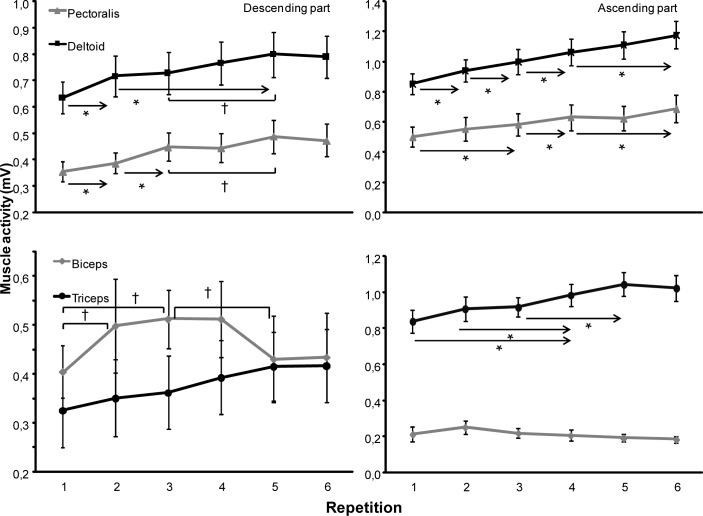
Mean (SD) root mean square (RMS) electromyographic activity for each repetition of the descending and ascending part in pectoralis major, anterior deltoid, biceps and triceps during 6 RM bench pressing. * indicates a significant difference between this repetition and all repetitions away from the sign, p ≤ 0.05 † indicates a significant difference between these two repetitions, p ≤ 0.05

**Figure 4.**
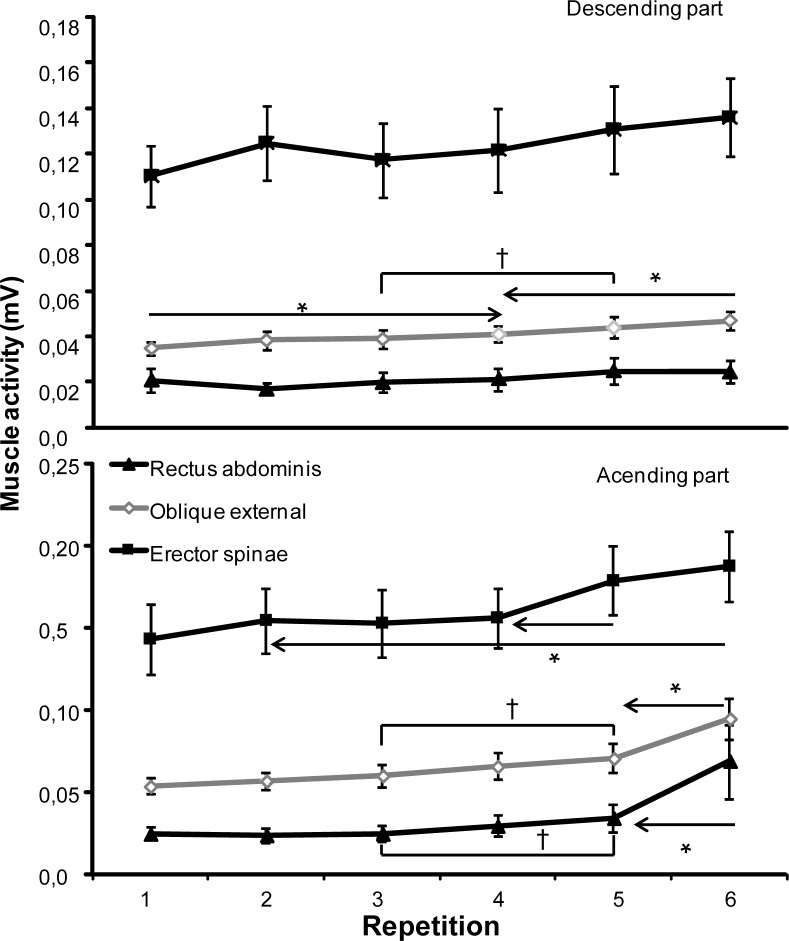
Mean (SD) root mean square (RMS) electromyographic activity for each repetition of the descending and ascending part in erector spinae, oblique external and rectus abdominis during 6 RM bench pressing. * indicates a significant difference between this repetition and all repetitions away from the sign, p ≤ 0.05 † indicates a significant difference between these two repetitions, p ≤ 0.05

For the EMG activity, in the descending part of bench press the repetition x muscle interaction was significantly different for the pectoralis, anterior deltoid and external oblique (F≥6.43, *p<*0.001; η^2^≥0.35) and not for the biceps, triceps, erector spinae and rectus abdominis (F≤2.38, *p*≥0.108; η^2^≥0.11). Post hoc comparison showed that the EMG activity of the deltoid and pectoralis muscles increased significantly from repetition 1 to 2. Furthermore, for the deltoid the muscle activity increased again from repetition 5, while for the pectoralis muscle the EMG activity increased from 2 to 3 and from 3 to 5 again (Figure 3). The biceps muscle activity increased significantly from repetition 1 to 2, with a stable activity towards the fourth repetition. As shown in Figure 3, the muscle activity, however, decreased significantly from repetition 3 to 5 to reach the same activity as in repetition 1. The activity of the oblique external increased significantly from repetition 1 to 4, 3 to 5 and again from 4 to repetition 6 (Figure 4). No significant differences were found for the triceps, rectus abdominal and erector spinae in the 6-RM test (Figures 3 and 4).

In the ascending part of bench press, the repetition x muscle interaction was significantly different for all the muscles (F≥4.76, *p*≤0.001; η^2^≥0.28) except the biceps (F=1.03, *p*=0.409; η^2^=0.08). Post hoc comparison showed that EMG activity of the deltoid increased significantly every repetition except between 4 and 5 (*p*=0.061) and between 5 and 6 (*p*=0.066). EMG of the pectoral muscles increased significantly from 1 to 3, 3 to 4 and from 4 to repetition 6 (Figure 3), while the triceps muscle activity increased significantly from 1 and 2 to 4 and from 3 to 5 (Figure 3).

The activity of the oblique external and rectus abdominal, only increased significantly in repetition 6 with the other and between repetition 3 and 5, while the erector spinae increased significantly in repetition 5 and 6 with previous ones (Figure 4).

## Discussion

The aim of this study was to examine the effect of fatigue during 6-RM bench pressing on muscle patterning and performance. As hypothesized, the total lifting time increased and the velocity in the ascending movement decreased. However, these kinematics were not found in the descending phase during the 6 repetitions of the bench press. Generally, the muscle activity increased during the six repetitions in the ascending movement, while only three of the seven measured muscles showed an increase over the six repetitions in the descending part of the 6-RM bench pressing.

As expected, decreased peak and average velocity of the barbell (Duffey and Challis, 2007; Sanchez-Medina and Gonzalez-Badillo, 2011) and increased lifting time (Duffey and Challis, 2007) in the ascending phase during the 6-RM occurred, indicating that fatigue occurred. However, in the descending phase the opposite was found: increased velocity and decreased lowering time. Especially from repetition 1 to 2 the total time decreased significantly due to a decreased lowering time of the barbell, while the lifting time increased (Figure 1). After repetition 2 the total time increased due to an increase in time in ascending part and constant descending part. In the last repetition, the time of the descending part also increased. The same occurred for the barbell velocity: the barbell velocity decreased only significantly from repetition 1 to 2 (higher descending velocity) and increased again only in repetition 6 (Figure 2). It indicates that repetition 1 and 6 significantly differ from the other four repetitions. In the ascending part, the velocity started to decrease from repetition 2 and continued in every repetition (Figure 2). This is most probably due to that at repetition 1, the participant had to elaborate the weight before he could increase the lowering velocity and thereby the total lowering time. In the next four repetitions the participant could control this velocity, while in the last repetition the increasing fatigue influenced the lowering velocity to avoid that the barbell stopped too late and the participant could not lift the barbell up again. An indication of this can be found in the muscle activation of the prime movers (triceps, pectoralis and deltoid muscles). The EMG activity increased from repetition 1 to 5 while there was similar EMG activity between repetition 5 and 6 in the descending part (Figure 3). The neural drive was probably at its maximum in repetition five for these muscles in the descending part.

The EMG activity in the prime movers was just the opposite of what Gentil et al. (2007) and Brennecke et al. (2009) found. They found similar EMG amplitude in the pectoralis and deltoid muscles, while an increase was found in the triceps during fatigue. This could be due to the inclusion of a pre-exhaustion protocol where the participants were fatigued by using 10-RM of the isolation chest press fly exercise targeting pectoralis and anterior deltoid respectively before bench pressing (Gentil et al., 2007; Brennecke et al., 2009). EMG between the pre exhaustion and the no exhaustion protocol were compared with each other. Thus, EMG activity was not compared within one set of repetitions. Therefore, the differences in findings of EMG activity of the prime movers can be explained by the protocol used in different studies (Gentil et al., 2007; Brennecke et al., 2009).

Duffey and Challis (2007) demonstrated that the velocity decreased and that the last repetition in sub maximal lifts resembled maximal 1-RM lifts. Thus, by fatiguing the muscles in the bench press, the load will relatively be heavier (higher % of 1-RM) and thereby the demand of the muscles would be larger. This was shown by Schick et al. (2010) who reported higher EMG activity of the pectoralis and deltoid muscles in lifts with 90% of 1-RM compared with lifts at 70% of 1-RM. Van den Tillaar and Ettema (2009) reported increased deltoid activity in 1-RM + 2.5kg compared with 1-RM lifts in the bench press, which is similar to our study on these muscles. Furthermore, the main velocity of the ascending part in our study decreased from 0.33±0.03 m/s in repetition 1 to 0.14±0.06 m/s in repetition 6, which is the same as in case of the bench press from 90% to 100% of 1-RM (González-Badillo and Sánchez-Medina, 2010). Thus, repetition 6 resembles maximal 1-RM lifting in the bench press. Sanchez-Medina and Gonzalez-Badillo (2011) suggested that the velocity loss was an indicator of neuromuscular fatigue and that lifting until exhaustion would give a large metabolic stress. In our study it indicated that this stress was also found in the ascending part of the lift in the prime movers (Figure 3) and trunk muscles (Figure 4), particularly in the trunk muscles repetition 6 significantly more antagonistic muscle co-activation was found (Figure 4).

As hypothesized, the EMG amplitude increased for the prime movers during the ascending phase (Figure 3). However, also the stabiliser muscle activity (i.e. abdominal and spine) increased over the 6 repetitions in the ascending phase during the 6-RM, especially in the last two repetitions (Figure 4) thereby indicating that fatigue also influenced these muscles within one series of lifts. The abdominal muscles activity increased significantly in the last repetition probably due to increased abdominal pressure to stabilize the trunk and maximize the force generation from the lower extremities. These processes are probably due to the maximal effort in the prime movers (Behm et al., 2005; Norwood et al., 2007; Santana et al., 2007). In contrast, the biceps muscle, also a stabilising muscle, was not influenced much during the 6-RM in the ascending phase. This indicates that the biceps is not so very active in the ascending movement since it is only an antagonist.

There are some limitations to our study. Firstly, the present study was limited by dividing the bench press only into two phases, which did not give detailed information about the activation pattern of the selected muscles during the whole bench press exercise. Secondly, only healthy resistance trained participants were recruited. Thus, the results cannot necessarily be generalized to other populations like elite power lifters. Thirdly, surface EMG can only provide an estimate of the neuromuscular activation, and that there is an inherent risk of crosstalk from neighboring muscles (Farina, 2006), even if a small inter-electrode distance was used. Lastly we only measured the EMG amplitude of the muscles and not the EMG median frequencies. Walker et al. (2012) showed that with sub maximal loading in the leg press EMG amplitude increases, but the median frequency decreases. Due to the limitations of the equipment used in our study, we were not able to measure the median frequencies of the muscles. In future studies this should be investigated to get a more detailed view about muscle behavior in the bench press during successive sets.

In conclusion, our study indicates that in a 6-RM bench press protocol performance decreases (lower barbell velocities and longer lifting times) and that the last repetition resembles maximal a 1-RM bench press. Furthermore EMG amplitude increases of the prime movers and the stabilizers (abdominal and spine), while the antagonist muscle (biceps) activity is not affected by fatigue during the lifting phase in 6-RM bench press.

The results of the two final repetitions demonstrated an important practical application for strength training athletes as fatigue increases dramatically. When performing only four repetitions at 6-RM load, strength training athletes maintain a high mechanical load, but decrease the metabolic stress and thus, create the possibility to maintain a high number of bench press sessions per week.
